# Stereotactic body radiation therapy as a salvage treatment for single viable hepatocellular carcinoma at the site of incomplete transarterial chemoembolization: a retrospective analysis of 302 patients

**DOI:** 10.1186/s12885-022-09263-3

**Published:** 2022-02-16

**Authors:** Sumin Lee, Jinhong Jung, Jin-hong Park, So Yeon Kim, Jonggi Choi, Danbi Lee, Ju Hyun Shim, Kang Mo Kim, Young-Suk Lim, Han Chu Lee, Hee Hyun Park, Jong Hoon Kim, Sang Min Yoon

**Affiliations:** 1 Department of Radiation Oncology, Asan Medical Center, University of Ulsan College of Medicine, 88, Olympic-ro 43-gil, Songpa-gu, Seoul, 05505 Republic of Korea; 2 Department of Radiology, Asan Medical Center, University of Ulsan College of Medicine, Seoul, Republic of Korea; 3 Department of Gastroenterology, Asan Medical Center, University of Ulsan College of Medicine, Seoul, Republic of Korea

**Keywords:** Hepatocellular carcinoma, Stereotactic body radiation therapy, Transarterial chemoembolization, Incomplete

## Abstract

**Background:**

To evaluate the clinical outcomes of patients who received stereotactic body radiation therapy (SBRT) for single viable hepatocellular carcinoma (HCC) at the site of incomplete transarterial chemoembolization (TACE).

**Methods:**

Patients treated with SBRT for single viable HCC after incomplete TACE between 2012 and 2017 at Asan Medical Center (Seoul, South Korea) were included. Incomplete TACE was defined as (1) evidence of viable HCC at the site of TACE on follow-up dynamic computed tomography (CT) or magnetic resonance imaging following one or more consecutive TACEs, (2) no definite tumor staining on superselective hepatic angiogram, or (3) no definite iodized oil uptake on post-embolization angiogram or CT. Doses of 10–15 Gy per fraction were given over 3–4 consecutive days. The primary outcome was local control rate at 3 years and secondary outcome included tumor response, overall survival rate, out-of-field intrahepatic recurrence-free survival, distant metastasis-free survival and treatment-related toxicities. Treatment-related adverse events were evaluated according to the common terminology criteria for adverse events, version 4.03.

**Results:**

A total of 302 patients were analyzed. The median follow-up duration was 32.9 months (interquartile range [IQR], 23.6–41.7) and the median tumor size was 2.0 cm (range, 0.7–6.9). The local control (LC) and overall survival rates at 3 years were 91.2 and 72.7%, respectively. 95.4% of the tumors reached complete response (CR) during the entire follow-up period (anyCR). The median interval from SBRT to anyCR was 3.4 months (IQR, 1.9–4.7), and 39.9 and 83.3% of the lesions reached CR at 3- and 6-months after SBRT, respectively. Radiation-induced liver disease was observed in 8 (2.6%) patients. No patients experienced gastroduodenal bleeding within the radiation field.

**Conclusion:**

SBRT could be considered a feasible salvage treatment option for HCC after incomplete TACE.

**Supplementary Information:**

The online version contains supplementary material available at 10.1186/s12885-022-09263-3.

## Background

Liver cancer is the fourth most common cause of cancer-related death worldwide, with hepatocellular carcinoma (HCC) being the most prevalent type of primary liver cancer [1, 2]. According to the updated Barcelona Clinic Liver Cancer (BCLC) staging system [3], transarterial chemoembolization (TACE) is recommended for patients with BCLC stage B disease based on the survival benefit demonstrated in randomized trials [4, 5]. Although hepatic resection or percutaneous ablative therapy are the currently recommended curative treatment options for patients with BCLC stage A disease, an analysis on actual practice pattern in clinics worldwide showed that TACE is also performed in patients with early-stage HCC because curative treatments are limited due to unresectable tumor location, portal hypertension, previous hepatectomy, and severe comorbidities [6].

Despite the clinical use of TACE in patients with early-stage HCC [7–9], there are still a number of incomplete necrosis within the treated tumor, i.e., incomplete TACE [10–12]. In this situation of a single viable tumor without multiple new lesions, salvage treatment options through effective subsequent local treatment modalities are required. However, only few studies to date have evaluated the effective local treatment option for residual HCC at the site of incomplete TACE [13, 14]. Therefore, current treatment guidelines for the management of HCC lack a specific recommendation on this issue.

Recent improvements in modern radiotherapy and imaging have permitted the delivery of optimal radiation doses to HCC while minimizing the amount of radiation to normal organs. Studies on stereotactic body radiation therapy (SBRT) for HCC demonstrated its efficacy for local tumor control from small- to large-sized HCCs and treatment-naïve to heavily-treated HCCs [15–17]. With growing evidence, recent guidelines have recommended SBRT as an alternative treatment option when curative treatment is not suitable [18–20]. However, the efficacy of SBRT as a local salvage treatment for residual viable HCC at the site of incomplete TACE is not well-known. Therefore, we evaluated the local control (LC) and other oncologic outcomes including response, survival, and toxicities of patients who received salvage SBRT for a single viable HCC at the site of incomplete TACE.

## Methods

### Definitions

HCC was diagnosed by typical findings, including intense arterial uptake followed by washout of contrast in the venous-delayed phase on dynamic computed tomography (CT) or magnetic resonance images (MRI) using a hepatocyte-specific contrast agent and/or pathologic confirmation according to the AASLD criteria [21]. Although the consensus on TACE failure/refractoriness has not been well established, this study focused on evaluating the efficacy of SBRT as a local salvage treatment for cases of ineffective response at the site of TACE without new recurrent lesions [22, 23]. In the present study, we defined incomplete TACE as follows: (1) evidence of a viable HCC at the site of TACE, which are typical diagnostic findings mentioned above, on follow-up dynamic CT or MRI after one or more consecutive TACE procedures, (2) no definite tumor staining on superselective hepatic angiogram, or (3) no definite iodized oil uptake on post-embolization angiogram or CT. Recurrent lesions at the margin of compact iodized oil uptake, which refers to complete response (CR) after the last TACE, were also included in this study. An example of incomplete TACE and viable tumor around the partial iodized oil uptake lesion is presented in Supplementary Fig. 1A. Tumor response was evaluated according to the modified Response Evaluation Criteria in Solid Tumors (mRECIST), which measures the viable tumor showing contrast enhancement in the arterial phase of dynamic CT or MRI [24]. The time point of the best response during the entire follow-up period was recorded and anyCR was defined as CR at the time point of the best response during the entire follow-up period. Radiation-induced liver disease (RILD) was defined as grade 2 or higher hepatic toxicity according to the Common Terminology Criteria for Adverse Events (CTCAE; version 4.03) or the worsening of Child-Pugh score to ≥2 in the absence of progressive disease within 3 months after SBRT. Local failure, out-of-field intrahepatic recurrence, and distant metastasis were defined as recurrence inside the planning target volume (PTV), recurrence within the liver but outside the treated lesion, and recurrent disease outside the liver, respectively.

### Patients

Eligibility criteria included single residual HCC with a Child-Pugh class A or B hepatic function; an Eastern Cooperative Oncology Group performance status (ECOG PS) score of 0–2; longest diameter of the HCC smaller than or equal to 7 cm; and an adequate residual functional liver volume (> 700 mL). Patients were excluded if they had macroscopic vascular invasion, extrahepatic metastases, double primary cancer, or a history of radiotherapy to the abdomen. This study protocol was approved by the Institutional Review Board of Asan Medical Center (IRB no. 2018-1004) and all methods were performed in accordance with the relevant guidelines and regulations. As this study was a retrospective analysis, a waiver of the requirement for informed consent was granted by the Institutional Review Board of Asan Medical Center.

### Transarterial chemoembolization

TACE was performed as described in previous studies [25–28]. Both superior mesenteric arteriography and common hepatic arteriography were performed to assess the overall anatomy, tumor burden, and portal vein patency. Cisplatin (2 mg/kg body weight) or adriamycin (50 mg) was infused using a microcatheter placed directly into selective catheterization of the feeding artery. An emulsion of iodized oil (lipiodol; Guerbet, Roissy, France) and cisplatin mixture was infused followed by embolization with Gelfoam slurry (Upjohn, Kalamazoo, MI, USA). If a residual tumor was observed at follow-up dynamic CT or MRI, on-demand subsequent TACE was performed 6–10 weeks after previous TACE. If compact lipiodolization without arterial enhancement was evident after TACE, regular follow-up with dynamic CT or MRI was performed in 2–3 months intervals at the physicians’ discretion.

### Radiotherapy

The simulation and target volume delineation procedures were the same as those described in our previous studies [14, 29]. Four-dimensional CT simulation using a 16-slice CT system (GE LightSpeed RT 16; GE Healthcare, Waukesha, WI, USA) was performed with free breathing. The CT images were sorted into 10 series according to the respiratory phase using 4D imaging software (Advantage 4D version 4.2; GE Healthcare). The gross tumor volume (GTV) included viable HCC and partial iodized oil uptake lesion representing initial HCC lesion with reference to diagnostic CT or MRI. For respiratory-gated radiotherapy, the internal target volume (ITV) containing tumor movement from 30 to 70% of the respiratory phase was delineated and 5-mm margins from the ITV were added for the PTV. SBRT planning was performed using a 3-dimensional radiotherapy planning system (Eclipse; Varian Medical Systems, Palo Alto, CA, USA) that used either multiple static conformal beams with energies of 6- or 15-MV photons or two arcs of volumetric-modulated arc therapy technique with a 10-MV flattening filter-free beam from a linear accelerator (TrueBeam STx; Varian Medical Systems). An example of the plan of SBRT is presented in Supplementary Fig. 1B. Over 95% of the PTV received 100% of the prescription dose and the chosen isodose covering PTV was between 85 and 90%, which was normalized to the center of the PTV. A total dose of 45–60 Gy in 3–4 fractions was planned and adjusted based on the dose recommended for preserving the liver function as follows: (1) the maximum dose allowed for 700 mL of the normal liver was 15 Gy in three fractions, and (2) the mean dose administered to the normal liver should not exceed 13 Gy in three fractions [30, 31]. Dose limitations for other critical organs included the following based on the Quantitative Analyses of Normal Tissue Effects in the Clinic [32]: (1) 2 cc of the esophagus or large bowel were limited to a total dose of < 21 Gy, (2) 2 cc of the stomach or duodenum were limited to a total dose of < 18 Gy, and (3) 2 cc of the spinal cord were limited to a total dose of 18 Gy. The actual beam delivery was performed with image guidance and a respiratory-gated beam delivery technique using an On-Board Imager (Varian Medical Systems). Image guidance was performed using cone-beam CT and gated fluoroscopy based on fiducial markers, residual iodized oil, surgical clips, or hepatic dome as a surrogate marker.

### Evaluation and statistical analysis

Assessments including physical examinations, laboratory tests, dynamic enhanced CT, and/or MRI were performed before and after SBRT at 2–3 month intervals. The primary outcome was local control rate at 3 years and secondary outcome included tumor response, overall survival (OS) rate, out-of-field intrahepatic recurrence-free survival, distant metastasis-free survival at 3 years. We also investigated the development of RILD described above and liver-related chronic toxicity. Overall survival, recurrence-free survival, and follow-up time were calculated from the date of starting SBRT to the date of death, recurrence, and last follow-up, respectively. For patients who underwent liver transplantation after SBRT, the transplantation day was defined as the last follow-up date and the data acquired afterward were censored for local tumor progression. Survival rates and time to best tumor response were calculated using the Kaplan-Meier method. Univariate and multivariate Cox proportional hazards models were generated to describe the association between the variables and LC or OS. Backward elimination Cox regression was utilized for multivariate analysis. Variables with *p* values < 0.2 in the univariate analysis were included in the multivariate analysis. The level of statistical significance was set at *p* values < 0.05. All statistical analyses were performed using the IBM SPSS Statistics for Windows, version 21 (IBM Corp., Armonk, NY, USA) and R software, version 3.6.3 (R Foundation for Statistical Computing, Vienna, Austria; http://cran.r-progect.org and web-r.org).

## Results

### Patient characteristics

**Fig. 1:**
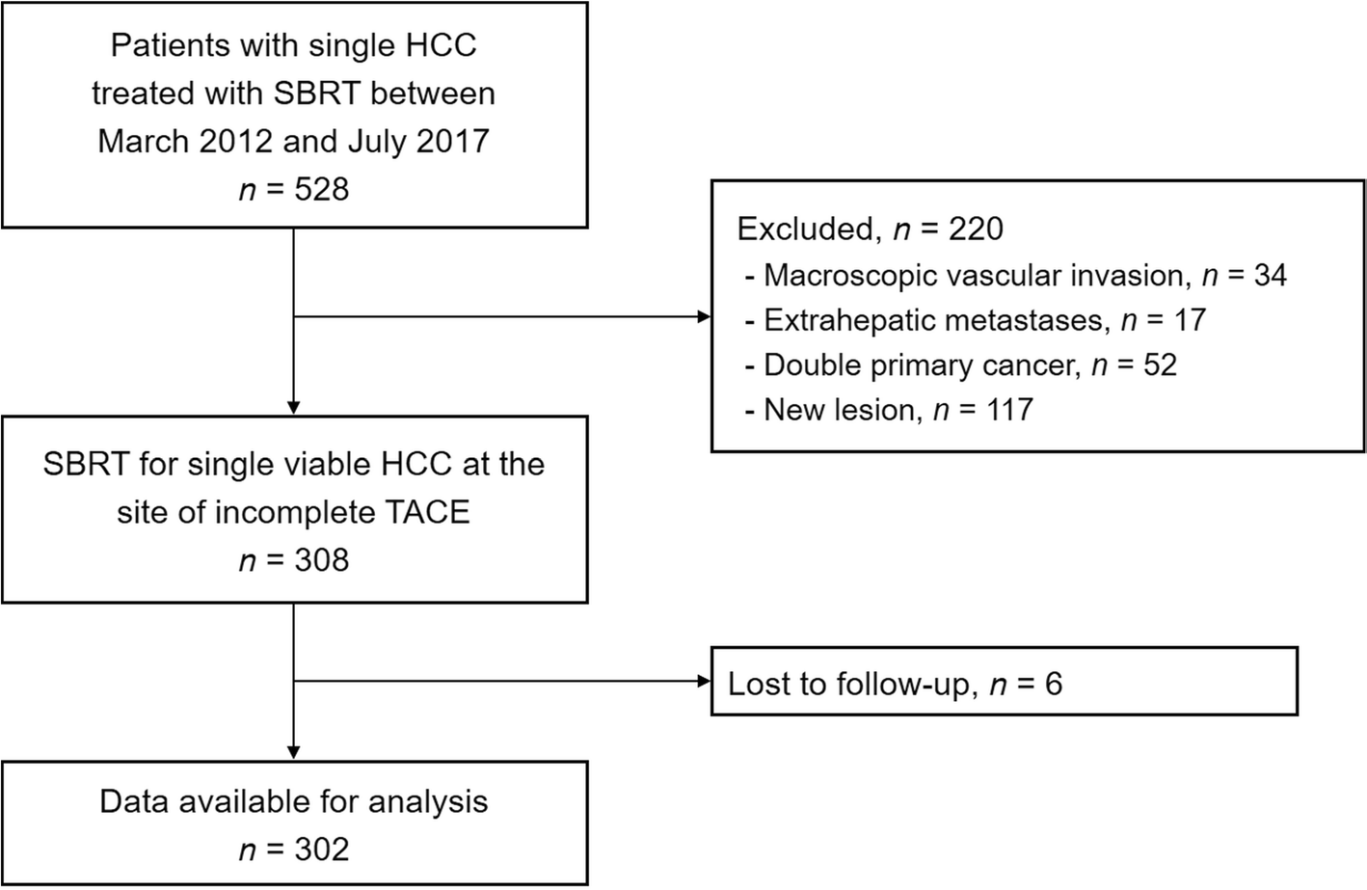
Flow diagram of the patients

Between March 2012 and July 2017, 528 patients were treated with SBRT for single HCC at Asan Medical Center. Among them, 302 patients who underwent SBRT for a single viable HCC at the site of incomplete TACE were included in the present study (Fig. 1). Table 1 summarizes the patient characteristics. The median age of the patients was 63 years (range, 37–90) and the majority of them were male (76.5%). The majority of the patients had ECOG PS of 0 (93.0%) and Child-Pugh class A hepatic function (91.4%). Until the last TACE, 73.5% of patients had multiple tumors, but only a single viable tumor remained after the last TACE. Most (84.4%) of the TACE were performed using cisplatin, iodized oil and cisplatin mixture, and gelatin sponge cubes. After the last TACE, CR, partial response (PR), stable disease (SD), and progressive disease was observed in 24.8, 20.9, 50.7, and 3.6% of the patients, respectively. The median tumor size was 2.0 cm (range, 0.7–6.9). The median radiation dose was 45 Gy (range, 36–60) with a median fraction size of 15 Gy (range, 12–20). The most common dose fractionation was 45 Gy in three fractions (88.4%), which corresponds to 113 Gy_10_ of biologically effective dose (BED; α/β = 10 Gy [33]) calculated using a linear-quadratic model.

**Table 1:** Patient characteristics

Variables	No. of patients (%) (*n* = 302)
Age, years, median (range)	63 (37–90)
Sex	
Male	231 (76.5)
Female	71 (23.5)
ECOG performance status	
0	281 (93.0)
1–2	21 (7.0)
Child-Pugh classification	
A	276 (91.4)
B	26 (8.6)
Viral etiology	
Hepatitis B virus	230 (76.2)
Hepatitis C virus	36 (11.9)
Non B, Non C	36 (11.9)
BCLC stage	
0	137 (45.4)
A	165 (54.6)
Tumor multiplicity at the last TACE	
Single	80 (26.5)
Multiple	222 (73.5)
Tumor size, cm, median (range)	2.0 (0.7–6.9)
Alpha-fetoprotein^a^, median (IQR)	9.2 (4.1–32.6)
≤ 20 ng/mL	203 (67.4)
> 20 ng/mL	98 (32.5)
Number of prior treatment sessions, median (IQR)	3 (1–5)
Prior treatments	
TACE only	159 (52.6)
TACE, RFA	60 (19.9)
TACE, PEI	2 (0.7)
TACE, RFA, PEI	8 (2.6)
Resection, TACE	46 (15.2)
Resection, TACE, RFA	25 (8.3)
Resection, TACE, PEI	2 (0.7)
Fractionation regimen, BED	
36 Gy/3fx (80 Gy_10_)	16 (5.3)
45 Gy/3fx (113 Gy_10_)	267 (88.4)
48 Gy/3fx (125 Gy_10_)	2 (0.7)
60 Gy/4fx (150 Gy_10_)	16 (5.3)
60 Gy/3fx (180 Gy_10_)	1 (0.3)

*Abbreviations*: *ECOG* Eastern Cooperative Oncology Group, *IQR* interquartile range, *BED* biologically effective dose

^a^Missing data in 1 patient

### Radiologic response

**Fig. 2:**
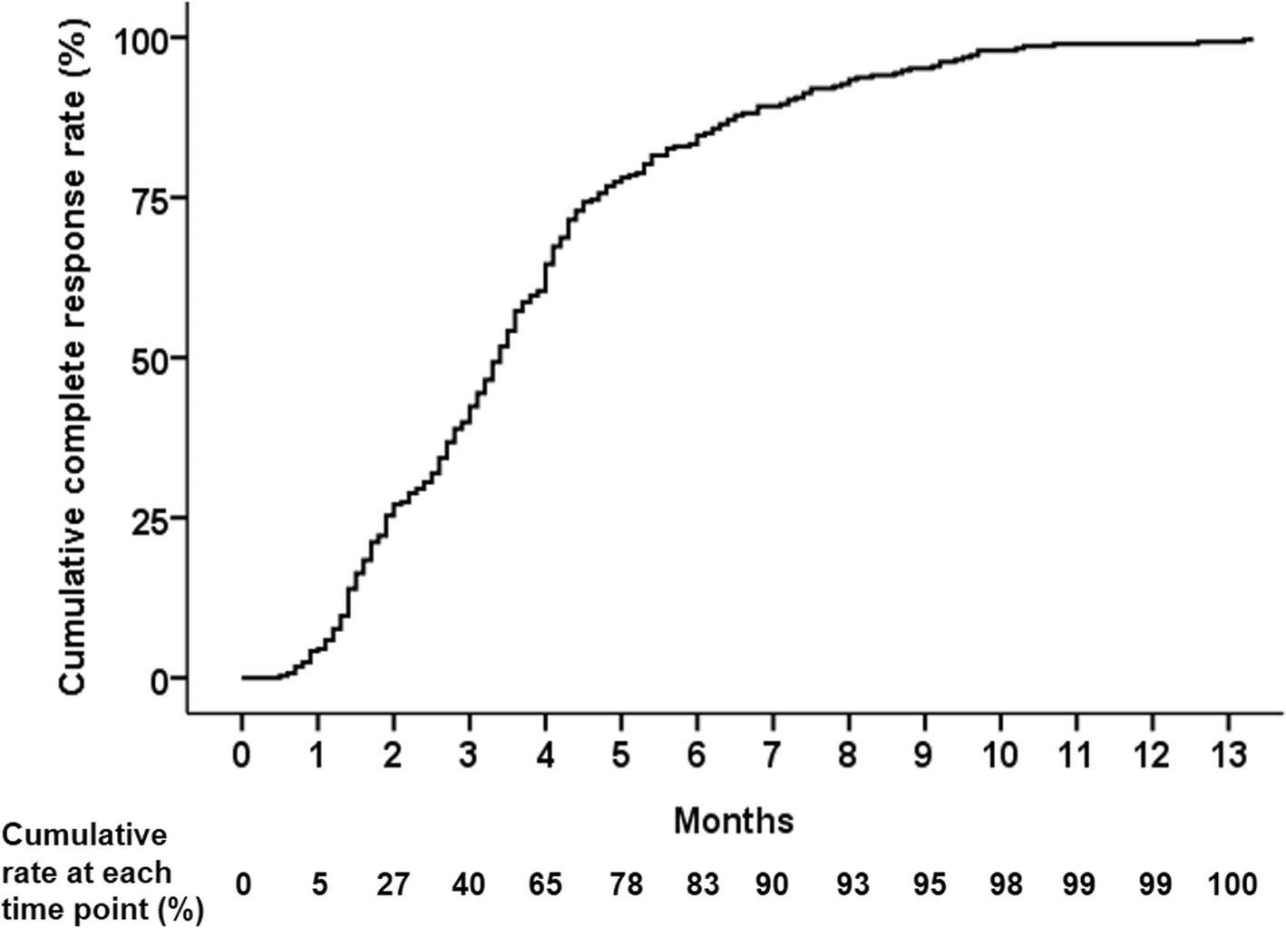
Cumulative complete response (CR) rate at each time point among patients who achieved anyCR

During the entire follow-up period, 288 lesions (95.4%) achieved CR; PR, SD, and progressive disease was noted in 2 (0.7%), 11 (3.6%), and 1 (0.3%) patients, respectively. Among the 288 CR lesions, 39.9 and 83.3% reached CR at 3 months and 6 months after completion of SBRT, respectively; 10% of CR was observed after 7.2 months of SBRT. The median interval from completion of SBRT to CR was 3.4 months (interquartile range [IQR], 1.9–4.7). The cumulative CR rate at each time point among patients who achieved anyCR is shown in Fig. 2. Only the tumor size significantly affected the anyCR in binary logistic regression (*p* = 0.006; hazard ratio, 1.83; 95% confidence interval, 1.19–2.83) (Supplementary Table 1). The average tumor size of the anyCR group and the non-anyCR group was 2.1 cm and 2.8 cm, respectively (*p* = 0.003). There was no statistically significant correlation between residual tumor burden after last TACE and anyCR after SBRT. However, the anyCR after SBRT in patients with progressive disease after the last TACE was lower than that in patients with marginal tumor recurrence around compact iodized oil uptake lesion (complete response by mRECIST) after the last TACE.

### Local control, recurrence-free and overall survival rates

**Fig. 3:**
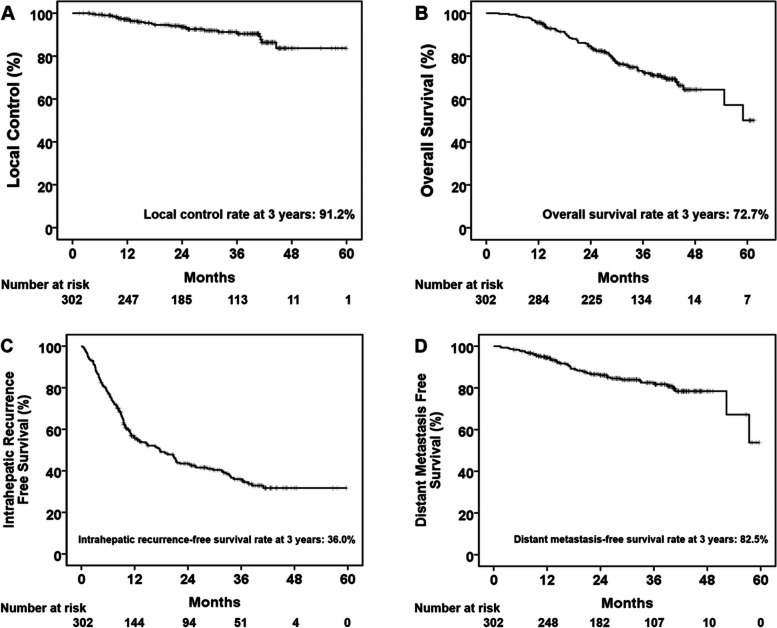
**A** Local control rates, **B** overall survival rates, **C** intrahepatic recurrence-free survival rates, and (D) distant metastasis-free survival rates of patients after stereotactic body radiation therapy

The median follow-up duration was 32.9 months (IQR, 23.6–41.7). Among 302 patients, 25 (8.3%) patients experienced local recurrence, resulting in a 3-year LC rate of 91.2% (Fig. 3A). In univariate analysis, BCLC stage, tumor size, BED, and anyCR were identified as significant prognostic factors for LC (Table 2). Multivariate analysis revealed that tumor size (*p* = 0.004) and anyCR (*p* < 0.001) were significant factors for LC. Among patients who experienced local tumor progression, eight patients had local tumor progression only, seven of whom received further salvage local treatment (6 TACE and 1 resection) and survived without recurrence; the remaining one patient had local tumor progression with portal vein tumor thrombosis (PVTT) and received palliative RT for the PVTT, but died with progressive disease.

**Table 2:** Univariate and multivariate analysis of prognostic factor for local control

Variables	Univariate		Multivariate	
	HR (95% CI)	*p* value	HR (95% CI)	*p* value
Age	0.99 (0.95–1.03)	0.513		–
ECOG PS		0.455		–
0	Reference			
1–2	0.47 (0.06–3.45)			
Child-Pugh class		-^a^		–
A				
B				
Etiology		0.322		–
HBV	Reference			
Others	0.58 (0.20–1.70)			
BCLC stage		0.013		0.290
0	Reference		Reference	
A	3.23 (1.29–8.09)		1.87 (0.59–5.91)	
Tumor size	1.82 (1.32–2.53)	< 0.001	1.78 (1.21–2.62)	0.004
Alpha-fetoprotein		0.376		–
≤ 20 ng/mL	Reference			
> 20 ng/mL	1.44 (0.65–3.20)			
Number of prior treatment sessions	1.07 (0.98–1.16)	0.156	1.01 (0.94–1.10)	0.735
BED	0.95 (0.92–0.98)	0.004	0.97 (0.94–1.00)	0.073
CR at 3 months		0.777		–
Yes	Reference			
No	1.13 (0.50–2.56)			
anyCR^b^		< 0.001		< 0.001
Yes	Reference		Reference	
No	22.48 (8.06–62.68)		15.58 (5.06–47.93)	

*Abbreviations*: *HR* hazard ratio, *CI* confidence interval, *ECOG PS* Eastern Cooperative Oncology Group performance status, *BED* biologically effective dose, *CR* complete response

^a^No valid analysis was performed because there was no local failure in the Child-Pugh class B group

^b^Achievement of complete response according to the mRECIST criteria at the time point of the best response during the entire follow-up period

The rates of OS, intrahepatic recurrence-free survival, and distant metastasis-free survival at 3 years were 72.7, 36.0, and 82.5%, respectively (Fig. 3B-D). Child-Pugh class A, BCLC stage 0, smaller tumor size, and higher BED were significant prognostic factors for OS in univariate analysis. Of these, Child-Pugh class A (*p* < 0.001) and higher BED (*p* = 0.014) were statistically significant prognostic factors for OS in multivariate analysis (Table 3). The achievement of CR at 3 months was not significantly associated with OS. Furthermore, there was no significant difference in the OS between those who had an early response (achievement of CR before 3 months) and those who had a late response (CR after 3 months) (Supplementary Fig. 2).

**Table 3:** Univariate and multivariate analysis of prognostic factor for overall survival

Variables	Univariate		Multivariate	
	HR (95% CI)	*p* value	HR (95% CI)	*p* value
Age	1.01 (0.99–1.03)	0.529		–
ECOG PS		0.375		–
0	Reference			
1–2	1.39 (0.67–2.89)			
Child-Pugh class		< 0.001		< 0.001
A	Reference		Reference	
B	3.98 (2.29–6.92)		3.13 (1.73–5.67)	
Etiology		0.819		–
HBV	Reference			
Others	1.06 (0.64–1.75)			
BCLC stage		0.021		0.860
0	Reference		Reference	
A	1.71 (1.08–2.69)		0.94 (0.49–1.80)	
Tumor size	1.29 (1.04–1.61)	0.021	1.26 (0.99–1.59)	0.059
Alpha-fetoprotein		0.089		0.341
≤ 20 ng/mL	Reference		Reference	
> 20 ng/mL	1.47 (0.94–2.30)		1.25 (0.79–1.98)	
Number of prior treatment sessions	1.04 (0.98–1.10)	0.209		–
BED	0.97 (0.95–0.98)	< 0.001	0.98 (0.96–1.00)	0.014
CR at 3 months		0.236		–
Yes	Reference			
No	1.33 (0.83–2.14)			
anyCR^a^		0.062		0.094
Yes	Reference		Reference	
No	2.38 (0.96–5.93)		2.23 (0.87–5.68)	

*Abbreviations*: *HR* hazard ratio, *CI* confidence interval, *ECOG PS* Eastern Cooperative Oncology Group performance status, *BED* biologically effective dose, *CR* complete response

^a^Achievement of complete response according to the mRECIST criteria at the time point of the best response during the entire follow-up period

### Toxicity

All patients received the planned SBRT without interruption. Elevation of transaminase or bilirubin levels of CTCAE grade ≥ 2, which may be associated with SBRT without progression of intrahepatic HCC, was observed in 8 (2.6%) patients. Five (1.7%) patients had a worsening of the Child-Pugh score ≥ 2 due to elevated bilirubin levels, but later showed improved or stable liver function during the follow-up period. One patient who received TACE and experienced asymptomatic biliary stenosis before SBRT developed hepatic failure and died 9.9 months after SBRT; however, the association between hepatic failure and SBRT was unclear. Asymptomatic biliary stricture in the central lesion after SBRT was found in 6 (2.0%) patients. In 11 (3.5%) patients, a rib fracture near the SBRT field was identified. There were no gastrointestinal complications such as bleeding or perforation associated with SBRT.

## Discussion

TACE was the most frequently used treatment modality for real-life management of patients with early- to advanced-stage HCC [6]. The current BCLC strategy recommends curative treatment options for patients with early-stage HCC with an expected median survival of 5 years or more [34, 35]. However, for patients who are not suitable for first-line therapy, TACE could be considered as a treatment option even though it is usually recommended for patients in more advanced stages in an approach known as the “stage migration strategy” [35]. In the BRIDGE study, which showed the management patterns for HCC in 14 countries, TACE was the second most commonly used treatment for HCCs with BCLC stage A. However, up to 57–77% of lesions still have viable tumor portions after TACE and thus require additional TACE or other treatment modalities [10–12]. In order to establish effective treatment changes in such cases, the Japanese guidelines revised the definition of TACE failure/refractoriness as (1) two or more consecutive ineffective responses within the treated tumors, (2) two or more consecutive progressions in the liver, (3) continuous elevation of tumor markers, (4) appearance of vascular invasion, and/or (5) appearance of extrahepatic spread [23]. In Japan, molecular-targeted therapy is recommended after TACE failure, especially for intermediate-stage HCCs [36, 37]. However, the consensus on TACE failure had not been well-established. Moreover, to date, only few studies have evaluated effective local treatment options for each situation of TACE failure, especially in early-stage HCCs [13, 14]. Therefore, current treatment guidelines for the management of HCC lack a recommendation on this issue.

Among the patterns of TACE failure and refractoriness, effective local salvage treatment should be considered in cases of ineffective response at the site of TACE without a new lesion, which was defined as incomplete TACE in the current study. We evaluated the clinical outcomes of patients who received salvage SBRT for a single viable HCC at the site of incomplete TACE, and found that SBRT had a high CR rate (95.4%), high LC rate (91.2% at 3 years), promising OS rate (72.7% at 3 years), and minimal SBRT-related toxicity.

There is a prospective randomized study that directly compared the TACE rechallenge and SBRT for patients with intermediate-stage HCC with incomplete response after TACE (NCT02323360). Preliminary analysis showed significantly higher local control of SBRT without any grade 3 toxicity [38]. As shown in the present study, favorable OS as well as excellent local control could be expected in a select group of patients. A prospective single-arm study by Takeda et al. reported the efficacy of SBRT for single HCC after TACE [39] by including patients with a single HCC less than 4 cm and TACE was omitted if embolization was difficult or if the patient refused. Of 90 patients, 32 patients were treatment-naïve, 58 received TACE, and 10 had a complete accumulation of iodized oil; in these patients, SBRT showed promising outcomes with a 3-year OS rate of 66.7% and a 3-year LC rate of 96.3%, which is in line with the results of the present study. SBRT was effective on liver-related cause-specific survival and OS regardless of whether TACE was performed. Therefore, if TACE for early-stage HCC is incomplete and other curative treatments are still not suitable, subsequent SBRT could be considered as a local salvage treatment.

In the present study, SBRT for small viable HCC at the site of incomplete TACE showed an excellent LC rate of 91.2% at 3 years. These results are in line with those of previous prospective and retrospective studies on SBRT for small HCC that reported high LC rates of 90–100% [14, 15, 29, 39–42]. As tumor responses after SBRT could be used as a surrogate measure for LC, rapid treatment changes can be considered if the tumor response is not sufficient. However, since the evaluation of the tumor response at a fixed time point according to mRECIST on the image after SBRT does not reflect the actual local control well, care should be taken not to determine the rapid treatment change with only the tumor response. According to Mendiratta-Lala [43, 44], even though there was no local progression in the treatment field, persistent arterial hyperenhancement in the MRI appeared up to 58% in the 3-6 months of evaluation after SBRT, which led to 75% of tumors being evaluated as SD. This hyperenhancement remained in 30% of the tumors even at 12 months. Only one recent phase II study that investigated the effect of SBRT on small HCC presented a reliable time point for determining tumor response [45]. The recent phase II study reported excellent oncologic results (5-year LC and OS rates of 97.1 and 77.6%, respectively) after SBRT for HCC, and also showed treatment response according to the mRECIST criteria at regular intervals (2-, 4-, 6-months after SBRT). Whereas the CR rate at 2 months was 30.2%, it was increased to 84.9% at 6 months after the completion of SBRT and eventually reported a local control rate of 100% at 2 years. The results of the present study are consistent with the phase II study; the median interval from completion of SBRT to the achievement of CR was 3.4 months (IQR, 1.9–4.7), and 39.9 and 83.3% of lesions reached CR at 3 months and 6 months after SBRT, respectively; 10% of the CR was observed 7.2 months after SBRT. The achievement of anyCR was significantly associated with LC, whereas CR at 3 months was not significantly associated with LC. There was no significant difference in the OS between those who had early response (achievement of CR before 3 months) and those who had late response (CR after 3 months). Based on these findings, it may be recommended that treatment changes be withheld for at least 6 months after SBRT if there is no definite local progression at the site of SBRT.

The study has the following limitations. First, the study has inherent biases due to its retrospective single-center study design. To compensate for this weakness, we included a large number of homogeneous patients who experienced ineffective responses at the site of TACE without a new lesion. Moreover, all patients were treated with a modern SBRT technique using respiratory-gated volumetric-modulated arc therapy. Second, the consensus on TACE failure had not been well-established and the small single HCC was the status at the time of SBRT, not the initial presentation. Finally, in order to focus on the efficacy of SBRT as a local salvage treatment, we only included incomplete TACE cases and not all TACE failure situations. Lastly, the follow-up period was not long enough to determine the 5-year survival. Therefore, long-term follow-up results are needed in further studies. Notwithstanding, we were able to fully capture the overall response during the entire follow-up period. Prospective randomized studies are warranted to confirm the benefit of salvage SBRT and to better define the group of patients that will benefit from the therapy.

## Conclusion

SBRT showed excellent clinical outcomes in terms of OS, LC, tumor response, and adverse events when used as an ablative treatment modality for single viable HCC at the site of incomplete TACE. SBRT could be considered for residual HCC after incomplete TACE. Treatment changes may be withheld for at least 6 months after SBRT to incomplete TACE site if there is no definite local progression at the site of SBRT.

## Supplementary Information

**Additional file 1: Supplementary Table 1.** Univariate and multivariate binary logistic regression analysis for the achievement of complete response during the entire follow-up period.

**Additional file 2: Supplementary Figure 1.** (A) An example of incomplete transarterial chemoembolization (TACE). Viable tumor around the partial iodized oil uptake lesion (red square) was observed on follow-up liver dynamic computed tomography images after incomplete TACE. (B) An example of a plan of stereotactic body radiation therapy. The lower dose limit for the color wash display was set to 80% of the prescription dose.

**Additional file 3: Figure 2.** Overall survival according to the timing of response after stereotactic body radiation therapy.

**Additional file 4.**

## Data Availability

All relevant data are within the paper and its Supplementary Dataset File. File name: supplementary_dataset_SBRT_Ineffective_TACE_302patients.xlsx.

## Abbreviations

HCC: Hepatocellular carcinoma
BCLC: Barcelona Clinic Liver Cancer
TACE: Transarterial chemoembolization
SBRT: Stereotactic body radiation therapy
LC: Local control
CT: Computed tomography
MRI: Magnetic resonance imaging
CR: Complete response
mRECIST: modified Response Evaluation Criteria in Solid Tumors
anyCR: complete response at the time point of the best response during the entire follow-up period
RILD: Radiation-induced liver disease
CTCAE: Common Terminology Criteria for Adverse Events
PTV: Planning target volume
ECOG PS: Eastern Cooperative Oncology Group performance status
GTV: Gross target volume
ITV: Internal target volume
OS: Overall survival
PR: Partial response
SD: Stable disease
BED: Biologically effective dose
IQR: Interquartile range
