# Supplementary material for: Stereotactic body radiation therapy as a salvage treatment for single viable hepatocellular carcinoma at the site of incomplete transarterial chemoembolization: a retrospective analysis of 302 patients
Source: BMC Cancer. 2022 Feb 16;22:175. doi: 10.1186/s12885-022-09263-3 (PMC8848650; doi:10.1186/s12885-022-09263-3)
Supplement: Supplementary file 1 — Additional file 1: Supplementary Table 1. Univariate and multivariate binary logistic regression analysis for the achievement of complete response during the entire follow-up period. [file 12885_2022_9263_MOESM1_ESM.docx]

| Variable | Univariate | | Multivariate | |
| --- | --- | --- | --- | --- |
|  | HR (95% CI) | *p* value | HR (95% CI) | *p* value |
| Age | 1.03 (0.97–1.09) | 0.376 |  |  |
| ECOG PS |  | 0.998 |  |  |
| 0 | Reference |  |  |  |
| 1–2 | 0.00 (0.00–8.9e+7457) |  |  |  |
| Child-Pugh class |  | 0.842 |  |  |
| A | Reference |  |  |  |
| B | 0.81 (0.10–6.45) |  |  |  |
| Etiology |  | 0.292 |  |  |
| HBV | Reference |  |  |  |
| Others | 1.83 (0.59–5.66) |  |  |  |
| BCLC stage |  | 0.206 |  |  |
| 0 | Reference |  |  |  |
| A | 2.15 (0.66–7.00) |  |  |  |
| Tumor size | 1.83 (1.19–2.83) | 0.006 | 1.86 (1.18–2.92) | 0.007 |
| Alpha-fetoprotein |  | 0.745 |  |  |
| ≤ 20 ng/mL | Reference |  |  |  |
| > 20 ng/mL | 0.82 (0.25–2.69) |  |  |  |
| mRECIST after the last TACE |  | 0.126 |  | 0.119 |
| Complete response^*^ | Reference |  | Reference |  |
| Partial response | 2.43 (0.22–27.40) | 0.474 | 1.93 (0.17–22.34) | 0.599 |
| Stable disease | 4.63 (0.58–37.20) | 0.150 | 4.41 (0.54–35.88) | 0.166 |
| Progressive disease | 16.44 (1.35–199.97) | 0.028 | 15.63 (1.26–194.59) | 0.033 |
| Interval from the last TACE to SBRT | 0.91 (0.77–1.08) | 0.289 |  |  |
| Number of prior treatment sessions | 1.08 (0.96–1.22) | 0.226 |  |  |
| BED | 0.97 (0.93–1.01) | 0.177 |  |  |
