# Supplementary figures and images for: Stereotactic body radiation therapy as a salvage treatment for single viable hepatocellular carcinoma at the site of incomplete transarterial chemoembolization: a retrospective analysis of 302 patients
Source: BMC Cancer. 2022 Feb 16;22:175. doi: 10.1186/s12885-022-09263-3 (PMC8848650; doi:10.1186/s12885-022-09263-3)

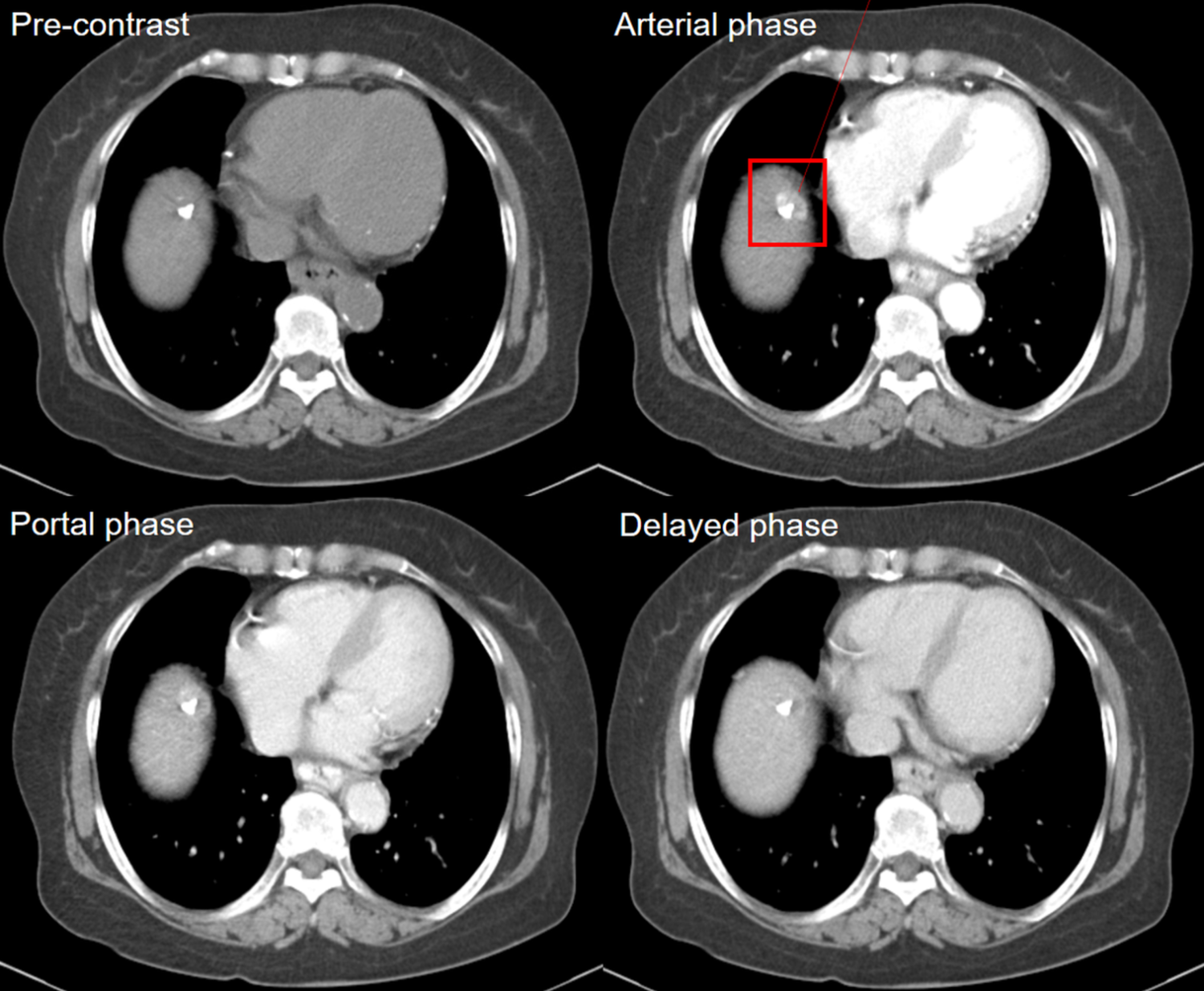

Supplement: Supplementary file 2 — Additional file 2: Supplementary Figure 1. (A) An example of incomplete transarterial chemoembolization (TACE). Viable tumor around the partial iodized oil uptake lesion (red square) was observed on follow-up liver dynamic computed tomography images after incomplete TACE. (B) An example of a plan of stereotactic body radiation therapy. The lower dose limit for the color wash display was set to 80% of the prescription dose. [file 12885_2022_9263_MOESM2_ESM.zip › Suppl_Figure_1A_new.tif]

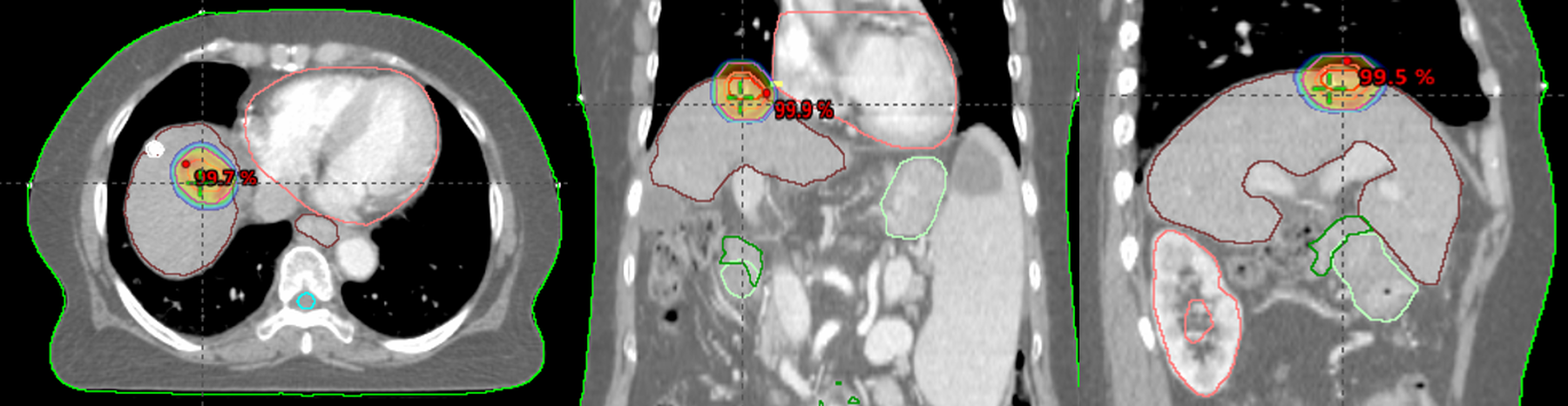

Supplement: Supplementary file 2 — Additional file 2: Supplementary Figure 1. (A) An example of incomplete transarterial chemoembolization (TACE). Viable tumor around the partial iodized oil uptake lesion (red square) was observed on follow-up liver dynamic computed tomography images after incomplete TACE. (B) An example of a plan of stereotactic body radiation therapy. The lower dose limit for the color wash display was set to 80% of the prescription dose. [file 12885_2022_9263_MOESM2_ESM.zip › Suppl_Figure_1B_new.tif]

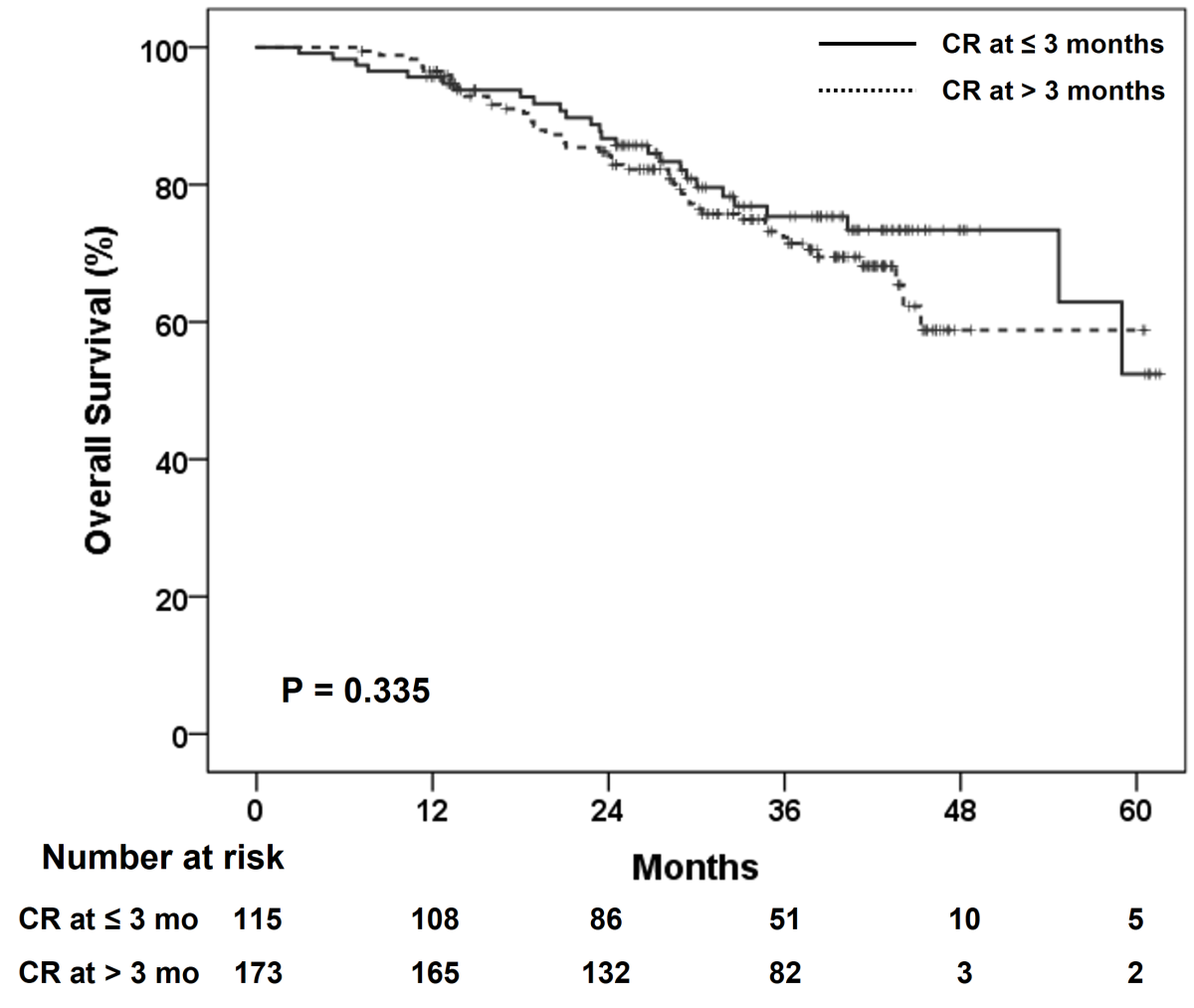

Supplement: Supplementary file 3 — Additional file 3: Figure 2. Overall survival according to the timing of response after stereotactic body radiation therapy. [file 12885_2022_9263_MOESM3_ESM.tif]
